# Early Prediction of Movie Box Office Success Based on Wikipedia Activity Big Data

**DOI:** 10.1371/journal.pone.0071226

**Published:** 2013-08-21

**Authors:** Márton Mestyán, Taha Yasseri, János Kertész

**Affiliations:** 1 Institute of Physics, Budapest University of Technology and Economics, Budapest, Hungary; 2 Oxford Internet Institute, University of Oxford, Oxford, United Kingdom; 3 Department of Biomedical Engineering and Computational Science, Aalto University, Aalto, Finland; 4 Center for Network Science, Central European University, Budapest, Hungary; Hungarian Academy of Sciences, Hungary

## Abstract

Use of socially generated “big data” to access information about collective states of the minds in human societies has become a new paradigm in the emerging field of computational social science. A natural application of this would be the prediction of the society's reaction to a new product in the sense of popularity and adoption rate. However, bridging the gap between “real time monitoring” and “early predicting” remains a big challenge. Here we report on an endeavor to build a minimalistic predictive model for the financial success of movies based on collective activity data of online users. We show that the popularity of a movie can be predicted much before its release by measuring and analyzing the activity level of editors and viewers of the corresponding entry to the movie in Wikipedia, the well-known online encyclopedia.

## Introduction

Living in the digital world of today, along with all the advantages also has its side effects and byproducts. Our daily life nowadays leaves a digital trace of all our activities in the recently developed Information and Communications Technology based environments. Our social communications through different digital channels, financial activities within e-commerce, physical locations registered by cell phone providers etc., are traced and recorded. In addition to such passive collection of data about online activity, we also actively share information about our feelings, emotional moods, opinions and views through the so called Web 2.0. or user generated content within social media. In addition to providing us with novel answers to classic questions about individual and social aspects of human life from scientific point of view, precise analysis of this huge amount of data can have practical applications to predict, monitor, and cope with many different type of events, from simple matters of daily life to massive crises in the global scale. For example, Sakaki et al. have developed an alerting system based on Tweets (posts in the Twitter microblogging service), being able to detect earthquakes almost in real time [1]. They elaborate their detection system further to detect rainbows in the sky, and traffic jams in cities [2]. The practical point of their work is that the alerting system could perform so promptly that the alert message could arrive faster than the earthquake waves to certain regions. Bollen et al. have analyzed moods of Tweets and based on their investigations they could predict daily up and down changes in Dow Jones Industrial Average values with an accuracy of 87.6% [3]. Saavedra et al. investigated the relationship between the content of traders' messages and market dynamics. They show that there is a positive correlation between the usage of “bundles” of positive and negative words with agents' overall financial performance [4]. Another example is using Twitter to predict electoral outcomes [5], however with its biases and limitations [6], [7]. Interesting studies have appeared treating the use of social media indicators to predict the scientific impact of research articles, e.g., short-term web usage (number of downloads from the pre-print sharing web site “arXiv”) [8] and Twitter mentions [9]. In a recent work, it is shown that Twitter mentions and arXiv downloads follow two distinct temporal patterns of activity, however, the volume of Twitter mentions is statistically correlated with arXiv downloads and early citations [10]. Preis et al. found a correlation between weekly transaction volumes of “S&P 500 companies” and weekly Google search volumes of corresponding company names [11]. By analyzing search queries for information about preceding and following years, a “striking” correlation between a country's GDP and the predisposition of its inhabitants to look forward is observed [12]. Based on Google search logs, Ginsberg et al. estimated the spread of influenza in the United States [13]. There are other examples of using social media streams to make predictions on news popularity in terms of the number of user-generated comments [14], [15] or the number of news visitors [16]. For a comprehensive literature review see [17].

Statistical analysis of motion picture markets has led to intriguing results, such as observing the evidence for a Pareto law for movie income [18], [19] along with a log-normal distribution of the gross income per theater and a bimodal distribution of the number of theaters in which a movie is shown [20]. By analyzing historical data about 70 years of the American movie market, Sreenivasan has argued that the movies with higher level of novelty (assigned based on keywords from the Internet Movie Database) produce larger revenue [21]. Despite much effort with different approaches, predicting the financial success of a movie remains a challenging open problem. For example, Sharda and Delen have trained a neural network to process pre-release data, such as quality and popularity variables, and classify movies into nine categories according to their anticipated income, from “flop” to “blockbuster”. For test samples, the neural network classifies only 36.9% of the movies correctly, while 75.2% of the movies are at most one category away from correct [22]. Joshi et al. have built a multivariate linear regression model that joined meta-data with text features from pre-release critiques to predict the revenue with a coefficient of determination 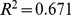 [23]. Since predictions based on classic quality factors fail to reach a level of accuracy high enough for practical application, usage of user-generated data to predict the success of a movie becomes a very tempting approach. Ishii et al. present a mathematical framework for the spread of popularity in society [24]. Their model, which takes the advertisement budget as an input parameter and generates a dynamic popularity variable, is validated against the number of blog posts on the particular movies in the Japanese Blogosphere. In other words they consider the activity level of bloggers as a representative parameter for social popularity. In an earlier work [25] a quantitative model based on “word of mouth” spreading mechanism was introduced in order to assess the quality of movies based on the “aggregated consumption data”. However, by analyzing the sentiment of blog stories on movies, Mishne and Glances emphasize that the correlation between pre-release sentiment and sales is not at an adequate level to build up a predictive model [26]. In a very interesting approach Asur and Huberman set up a prediction system for the revenue of movies based on the volume of Twitter mentions [27]. They achieve an adjusted coefficient of determination of 0.97 on the night before the movie release for the first weekend revenue of a sample of 24 movies. In a later work, however, Wong et al. show that Tweets do not necessarily represent the financial success of movies [28]. They consider a sample of 34 movies and compare the Tweets about the movies to evaluations written by users of movie review web sites. They argue that predictions based on social media could have high precision but low recall. Yun and Gloor showed that the betweenness centrality of a movie in a network representation of its presence on the Web is correlated with its financial success [29]. In a rather novel approach, Oghina et al. have made use of Twitter and YouTube activity streams to predict the ratings in the Internet Movie Database (IMDb), which is among the most popular online movie databases [30].

Wikipedia, as a predominant example of user-generated media, has been intensely studied from different points of view. Its size and growth [31]–[33], topical coverage and notability of entries [34]–[36], conflict and editorial wars among users [37]–[41], editorial patterns [42] and linguistic features [43] are only few examples of research topics associated with Wikipedia. We are aware of two comprehensive reviews [44], [45] and a brief hands-on guide to some of the most recent Wikipedia research [46].

Although effects of external events on the activity of Wikipedia editors [47], [48] and the number of page views [49], [50] have been studied in detail, usage of Wikipedia as a source of information to detect and predict events in real world has been limited to the work by Osborne et al. [51], in which they used Wikipedia page views to fine-filter the outcome of their algorithm for Twitter-based “first story detection” and a very recent work by Georgescu et al., in which Wikipedia edits are introduced as “entity-specific news tickers and time-lines” generators [52]. And finally in an interesting work published later than the first revision of the current manuscript, Moat et al. reported on the predictive power of Wikipedia data for financial fluctuations [53].

In this work we consider both the activity level of editors and the number of page views by readers to assess the popularity of a movie. We define different predictor variables and apply a linear regression model to forecast the first weekend box office revenue of a set of 312 movies, which were released in the United States in 2010. Our analysis not only outperforms the previous works by the much larger number of movies we have investigated, but also improves on the state of the art by providing reasonable predictions as early as one month prior to the release date of the movie. Finally, our statistical approach, free of any language based analysis, e.g., sentiment analysis, can be easily generalized to non-English speaking movie markets or even other kinds of products.

## Results

**Figure 1:**
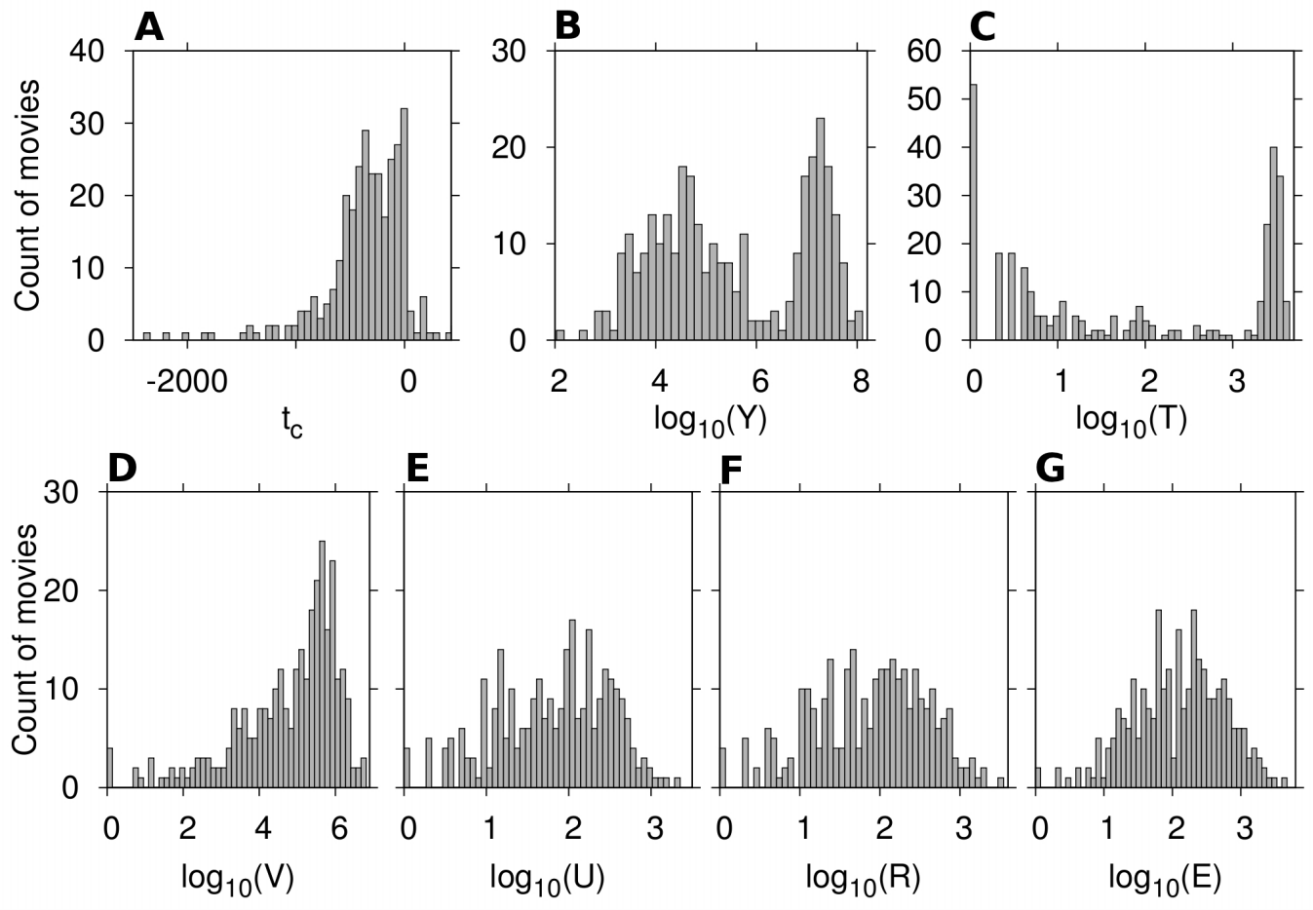
Histograms of different variables for our sample of 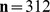 movies from 2010. A: Time of creation 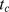 of the corresponding article in Wikipedia, shown in days of *movie time* (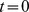 is the release time), B: Release weekend box office revenue in the U. S., in USD C: *number of theaters* that screened the movie on the first weekend, D: Accumulated *number of views*, and E: *users*, F: *edits*, G: *rigor* for the Wikipedia page up to 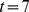 days after release.

According to data from Box Office Mojo, there were 535 movies that were screened in the United States in 2010 (see the Methods section). We could track the corresponding page in Wikipedia for 312 of them. A closer look at the history of these 312 articles shows that many of them are created a lot earlier than the release date of the movie (Fig. 1(A)). This enables us to follow the popularity of the movie much in advance. To estimate the popularity, we followed four activity measures; 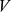: *Number of views* of the article page, 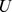: *Number of users*, being the number of human editors who have contributed to the article, 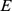: *Number of edits* made by human editors on the article, and 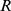: *Collaborative rigor* (or simply *rigor* [54]) of the editing train of the article. To have a consistent time framework, we set the release time of the movie as 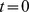. For more details see the Methods section. Examples of the daily increments of number of views and number of users are shown in Fig. S1. The daily increments of both variables rise and fall around the day of release similarly to observations by Ishii et al. [24]. In addition to these, an essential parameter for predicting the movie revenue is *the number of theaters* that screen the movie 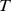, which is included in our set of parameters. The complete dataset including the financial data as well as Wikipedia activity records is available via Dataset S1. To have an overall image of the sample, histograms of the accumulated values of the 4 activity parameters from the first edit on the article up to 7 days after release, along with the first weekend box office revenue, and the number of theaters screening the movie are depicted in Fig. 1(B–F). It is clear that revenues among the sample have a bimodal distribution (Fig. 1(B)). This is in accord with [20], where authors report that the distribution of the total revenue of a sample of 5,222 movies released over the period of 1999–2008 across theaters in the USA, exhibits bimodal nature and have been fit using a superposition of two log-normal distributions. It also shows that Wikipedia coverage is not limited to financially successful movies. The considerable amount of activity on Wikipedia articles (Fig. 1(D–G)) indicates the richness of the data. However, before building a regression model, the correlations between the activity parameters and the box office revenue should be examined first.

**Figure 2:**
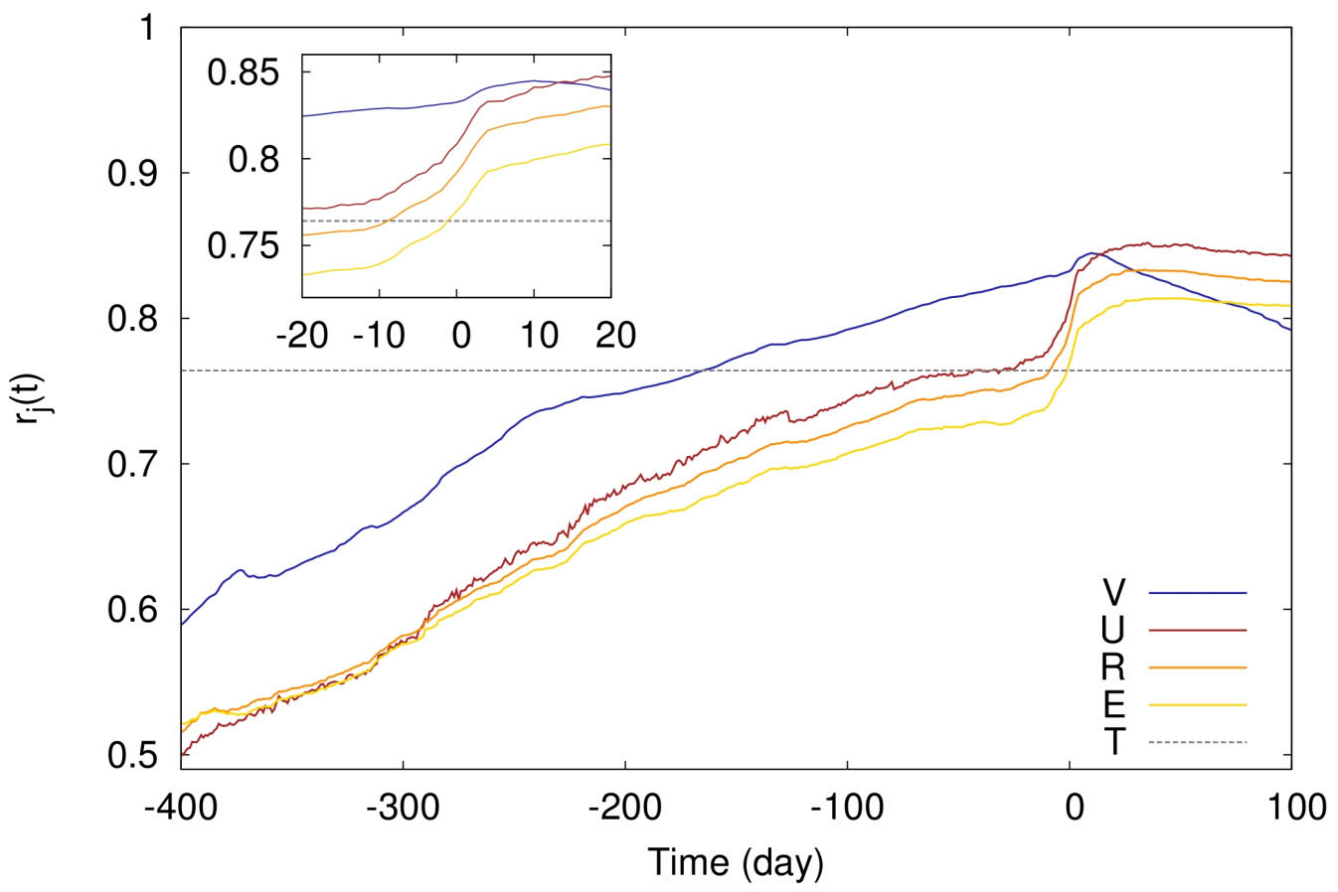
Temporal evolution of 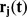, the Pearson correlation of the box office revenue with different predictors. The shorthands 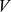, 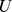, 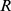, 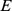, and 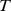 denote the *number of views*, the *number of users*, the *rigor*, the *number of edits*, and the *number of theaters*, respectively. Time is measured in movie time. *Inset*: magnified detail of the main panel, showing the Pearson correlation around the day of release. Dashed horizontal line shows the correlation for *the number of theaters*.

The Pearson correlation coefficient 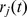 between the accumulated value 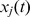 of the 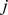-th predictor variable from the inception of the article up to time 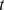 before the movie release and the box office revenue 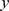 is calculated as
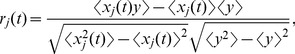
(1)with 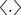 indicating average over the whole sample. Temporal correlations are shown in Fig. 2. For all activity based predictors the correlation coefficient gradually increases as time approaches the day of release and around the day of release, correlation suddenly rises. Note that 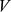 shows the highest correlation with the revenue prior to the release pf movies.

**Figure 3:**
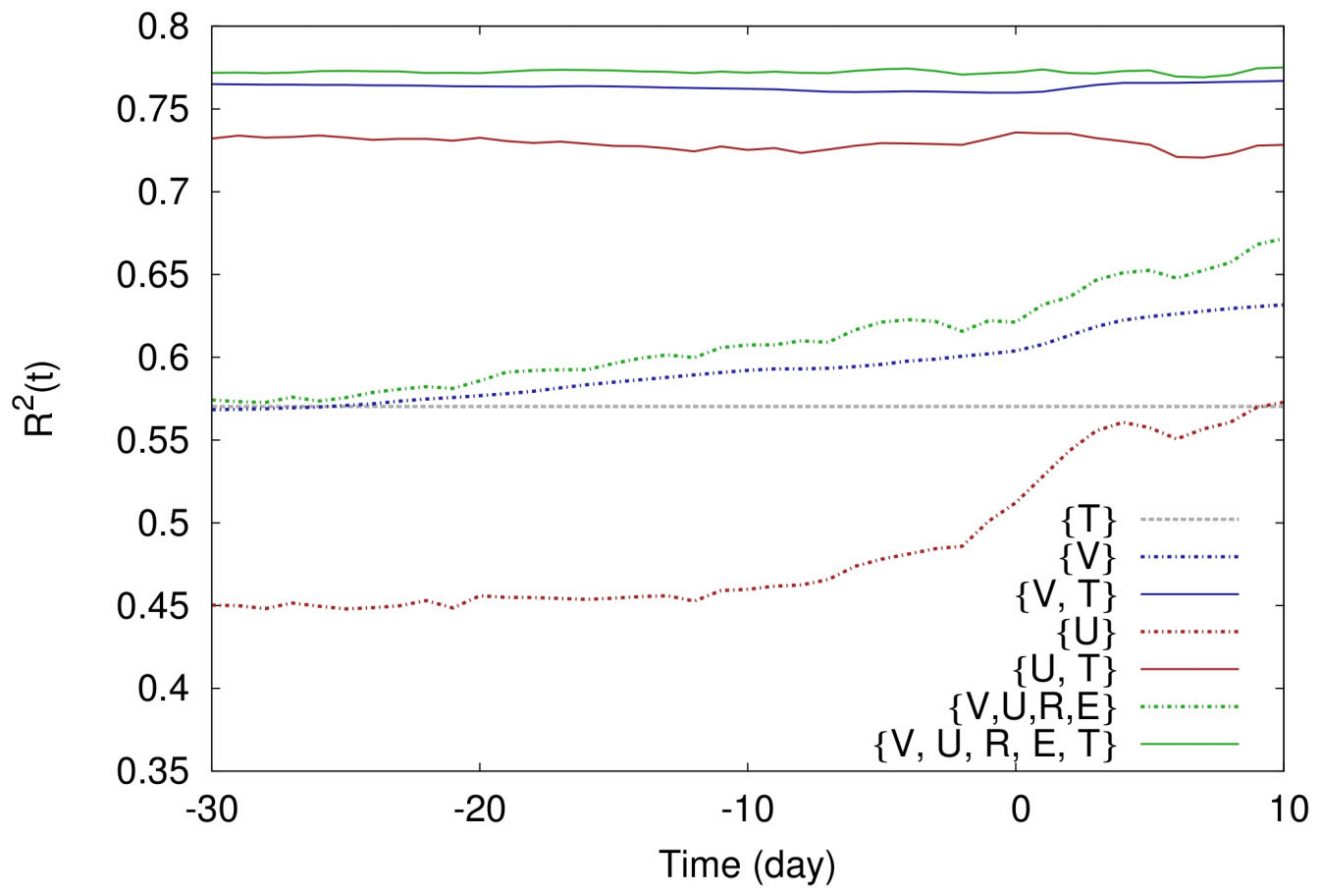
Coefficient of determination of the multivariate linear regression model fed by different set of input variables. The shorthands 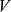, 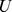, 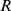, 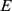, and 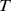 denote the *number of views*, the *number of users*, the *rigor*, the *number of edits*, and the *number of theaters*, respectively. The coefficient of determination was calculated using 10-fold cross-validation (see the Methods section). The dashed gray line shows the coefficient of determination for linear regression solely based on the *number of theaters*.

We build a multivariate linear regression model for predicting the box office revenue 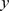. The general form of a regression model at time 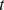 before release, based on a set of predictor variables 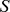 is
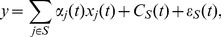
(2)where 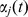
s are time varying parameters of the linear regression model, 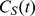 is a constant and 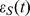 is the noise term. We feed the model with different combinations of predictor variables and characterize the goodness of different sets by calculating the coefficient of determination 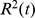. The coefficient of determination is calculated using 10-fold cross-validation (See Methods section). Temporal evolution of 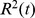 is shown for different predictor sets 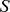 in Fig. 3. While a model employing 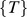 can be seen as a benchmark of the state of the art in real market predictions, the model solely fed by 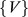 predicts roughly as well as that. Combinations of 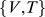 and 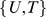 score well above the benchmark indicating the relevance of activity measures for prediction. Among all sets considered (not shown here), 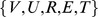 yields the highest coefficient of determination, which reaches 0.77 around a month before the movie release.

## Discussion

Results presented above clearly show how simple use of user generated data in a social environment like Wikipedia can enhance our ability to predict the collective reaction of society to a cultural product. While these results can be of practical application for marketing purpose, especially in combination with other source of information, our main aim is to demonstrate the extent of engagement of members of the public in the peer-production platforms. The introduced approach can be easily generalized to other fields where mining of public opinion provides valuable insights, e.g., financial decisions, policy making, and governance. We believe that Wikipedia and similar mass-collaboration platforms can serve as alternative resources for social media streams with higher level of professionalism and deeper engagement of users. Since the methods presented here are independent of the language of the medium, they can be easily generalized to other languages and local markets.

It is worth mentioning that to feed our predictive model, we have tried several other activity measures, which can potentially be predictive parameters, e.g., time span between the creation of the article and the release time and length of the article. However these quantities did not show any significant correlation with the box office revenue and consequently were excluded from the model.

**Figure 4:**
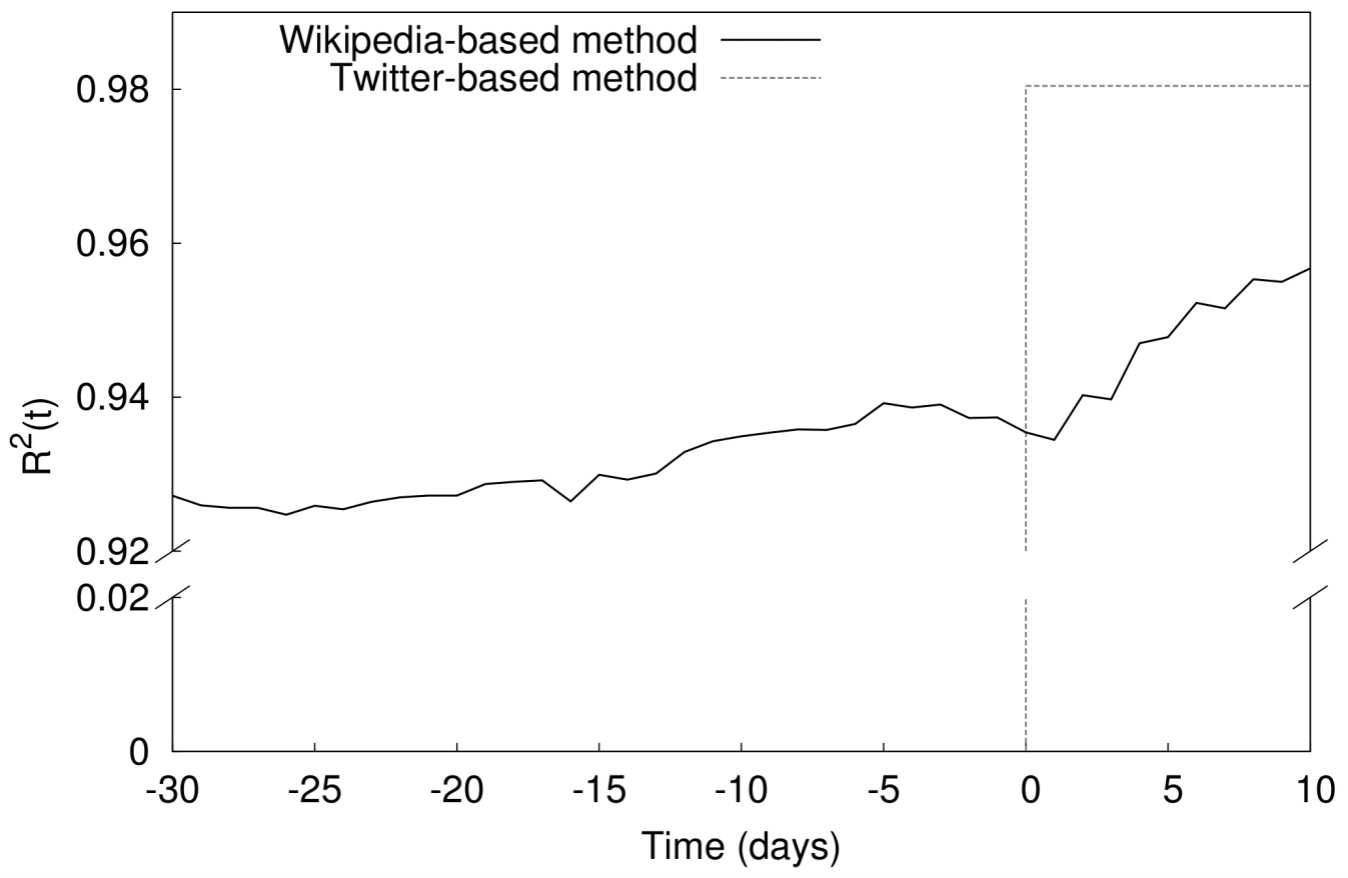
Comparison of the results with the Twitter-based prediction in Asur and Huberman work [27]. Same sample of 24 movies is considered as both training and test set. The coefficient of determination obtained with the Twitter-based method is 0.98 at the night of the release (day 0 in movie time).

We also compare the predictive model based on Wikipedia activity measures with the results of the Twitter-based model provided in the 2010 study of Asur and Huberman [27]. Asur and Huberman use a sample of 24 movies to train and test their model. In the same approach we train and test our model focusing on the same set of movies. The 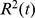 of our Wikipedia model reaches 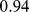 few days before release, while it is 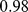 for the Twitter model. However, the results of the Twitter study are limited to the night before release, while the analysis presented here can make predictions with reasonable accuracy (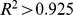) as early as one month before release (See Fig. 4). One should also bear in mind that the Wikipedia model does not require any complex content analysis and only relies on statistical measures of activity level. The predicting power of the Wikipedia-based model, despite its simplicity compared to the Twitter, can be explained by the fact that many of the Wikipedia editors are committed followers of movie industry who gather information and edit related articles significantly earlier than the release date, whereas the “mass” production of tweets only occurs very close to the release time, mostly evoked by marketing campaigns.

**Figure 5:**
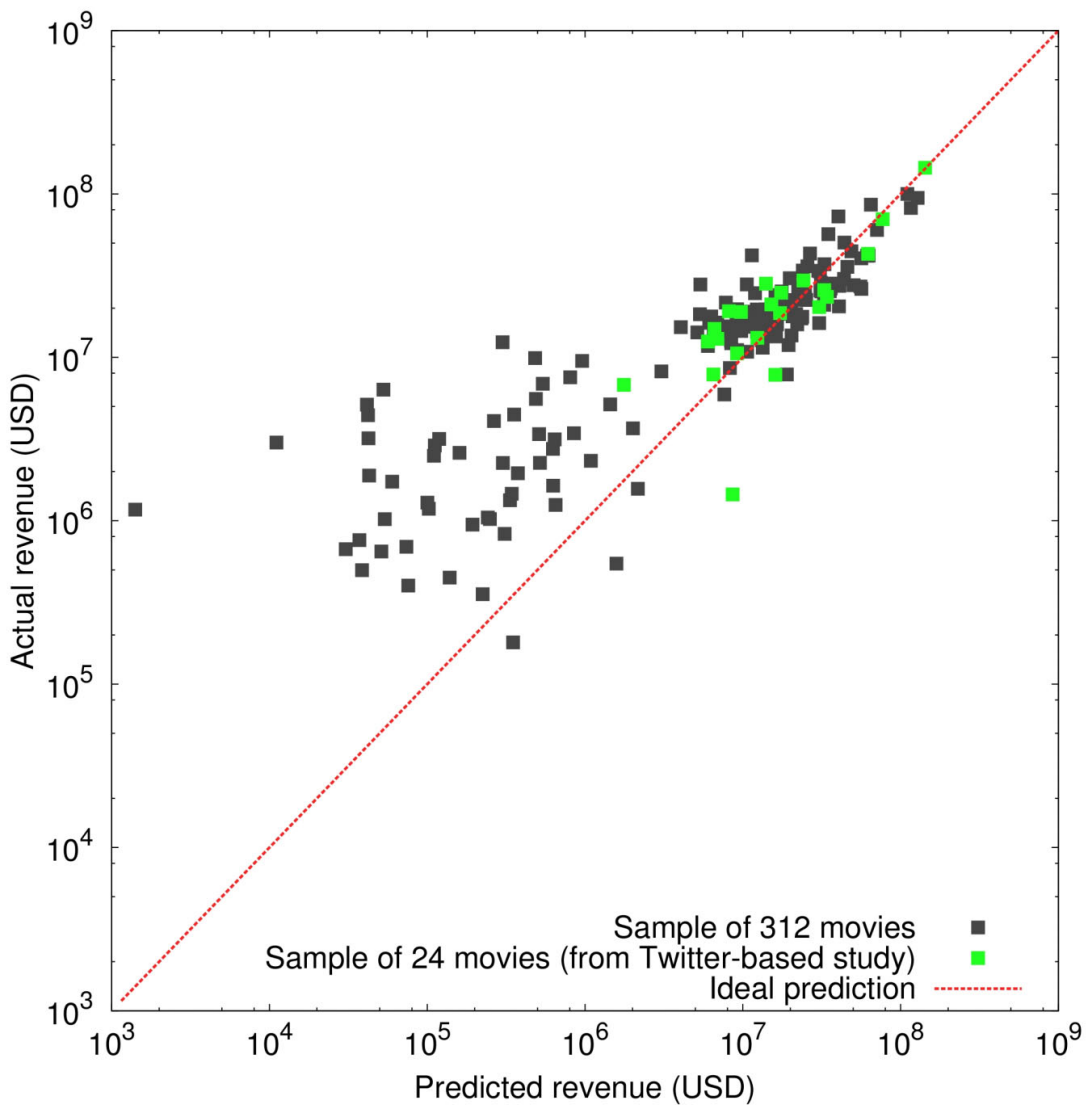
First weekend box office revenue in the U. S. against its predicted value by the Wikipedia model at 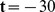 days. Green dots are representing the smaller sample of 24 movies common in Twitter and Wikipedia studies, and black dots are movies from the 2010 sample of 312 movies. Note that negative predicted revenues for some of the very unpopular movies could not be shown in the logarithmic scale.

Fig. 5 shows the actual revenue of movies in the sample against the predicted revenue at 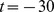 days. It is evident that the prediction is more precise for more successful movies. When less successful movies are considered, deviations from the diagonal line denoting perfect prediction, increase. Some examples of the movies whose box office receipts were predicted accurately are *Iron Man 2*, *Alice in Wonderland*, *Toy Story 3*, *Inception*, *Clash of the Titans*, and *Shutter Island*. However, the model failed to provide accurate predictions for less successful movies, e.g., *Never Let Me Go*, *Animal Kingdom*, *The Girl on the Train*, *The Killer Inside Me*, and *The Lottery*. This systematic difference in precision can be explained by the amount of data available for each class of movies. Clearly the model works more accurately when the movie is more popular and the volume of the related data is larger. By considering the green squares which represent the movies in the sample predicted by the Twitter model, one realizes that most of the movies predicted by the Twitter method are among the successful ones, therefore applicability of the Twitter model on movies with medium and low popularity levels remains an open question.

While we tried to keep our model as simple as possible and based on only a few variables, one could possibly enhance the efficiency of prediction by applying more sophisticated statistical methods, such as neural networks on more detailed content-related parameters e.g., the controversy measure of the article [38].

## Methods

In this study we consider a sample of 312 movies, which were released in the United States in 2010. The complete dataset including the financial data as well as Wikipedia activity records is available via Dataset S1. To obtain this dataset, first the list of 2010 movies distributed in the U. S. is acquired from Box Office Mojo (http://boxofficemojo.com) along with their accompanying financial data (535 movies). Financial data consist of the opening weekend box office revenue and the number of theaters screening the movie.

In order to locate the corresponding articles in Wikipedia, we use the category system of Wikipedia. Wikipedia articles are classified into one or more categories by users. We match the title of the movies in the Mojo database with the title of Wikipedia pages in categories 2009 films and 2010 films. Inclusion of the category 2009 films is necessary because of movies that were released in 2010 in the U.S. but which could have already entered the international market during 2009, and hence were classified in the category 2009 films in Wikipedia. To achieve the best possible match of the titles, they were stripped of punctuation and postfixes. Wikipedia uses the latter to maintain the uniqueness of every title, such as in the case of Avatar (2009 film) and Avatar (computing). As a result of the matching process described above, a sample consisting of the financial data and the corresponding Wikipedia page for 312 movies was obtained.

For the sake of convenience we introduce *movie time*, a common time coordinate for the movies in the scope of our study. By definition, movie time is measured from the time of release in the U.S. All temporal variables are measured in movie time. Throughout this study, we consider accumulated values of parameters from the inception of the article to the prediction time 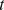 for each activity measure. The four activity measures are defined as the following:

*Number of users*, 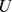: the number of different human users who contributed to the page.

*Number of edits*, 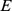: the number of modifications made by human users on the article.

*Collaborative rigor*, 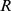: similar to the number of edits; however it counts multiple subsequent edits by the same user as one edit [54]. It avoids counting multiple edits by the same user in a short period, e.g., to correct errors in their previous contribution.

**Figure 6:**
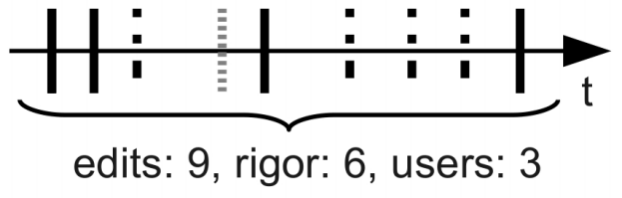
Illustration of different variables characterizing the activity of Wikipedia editors on an article. Each tick on the axis represents a modification of the page. Different tick styles refer to different users.

A schematic illustration of these activity measures is presented in Fig. 6. These three variables are calculated using the page history databases of Wikimedia Toolserver (http://toolserver.wikimedia.org), which register information about every modification made to the pages of Wikipedia. To ensure that the above variables count solely human activity, contributions made by *bots* are excluded from calculations. Bots are automated scripts which facilitate automatic tasks such as spell checking. Contributions made by bots are registered in the same way as revisions by humans; however, they can be distinguished from human activity by noting a special entry in the databases of Wikimedia Toolserver, called the *bot flag*.

*Number of views*, 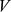: the number of times a given page is viewed from its inception up to the time 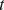. This data is extracted from the page view statistics section of the Wikimedia Downloads site (http://dumps.wikimedia.org/other/pagecounts-raw) through the web-based interface of “Wikipedia article traffic statistics” (http://stats.grok.se). Wikimedia Downloads counts views only since December 2007 and the view count data for July 2008 is corrupted. Therefore it is impossible to count the exact total number of views till the time of prediction for all considered pages. We have counted the page hits from 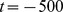 days before release, which according to Fig. 1(A), is sufficiently early. Another challenge is created by the renaming of the articles, which splits page hit counts into subsets according to the various titles the page possesses throughout its history. To cope with this problem, we followed the logs of “title moves” in the article history to track back and merge the whole page hits. Note that in the the dataset there are records on Wikipedia page requests for non-existing pages as well, which give us an indicator of the public interest in a movie even before its Wikipedia article is created and therefore we did not exclude such records from the data. *Number of theaters*: the count of movie theaters that screen the movie on the first weekend of its release.

To calculate the coefficient of determination, we carry out 10-fold cross-validation by randomly dividing our sample of 2010 movies into 10 subsets first. In the next step the model is trained for the union of the 9 subsets and tested on the remaining 10th subset. This is repeated for all 10 permutations of the subsets and the coefficient of determination for the model is obtained as the average over the permutations.

## Supporting Information

Figure S1
**Temporal evolution of Wikipedia-based predictors for two individual movies: The Wolfman (2010) and MacGruber.** The daily increments of *number of views* 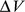 and *number of users* 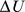 are shown for the articles in English Wikipedia that correspond to the two movies. The temporal axis shows movie time, i.e., a time-frame in which 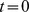 corresponds to the release date. The Wolfman earned a box office revenue of $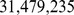 on the release weekend while MacGruber gained only $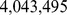. Accordingly, predictor variables take larger values in the case of The Wolfman.(TIFF)

Dataset S1 The dataset under study, including the financial and Wikipedia activity data is also available at http://wwm.phy.bme.hu/SupplementaryDataS1.zip.
(ZIP)
