# Supplementary figures and images for: Early Prediction of Movie Box Office Success Based on Wikipedia Activity Big Data
Source: PLoS One. 2013 Aug 21;8(8):e71226. doi: 10.1371/journal.pone.0071226 (PMC3749192; doi:10.1371/journal.pone.0071226)

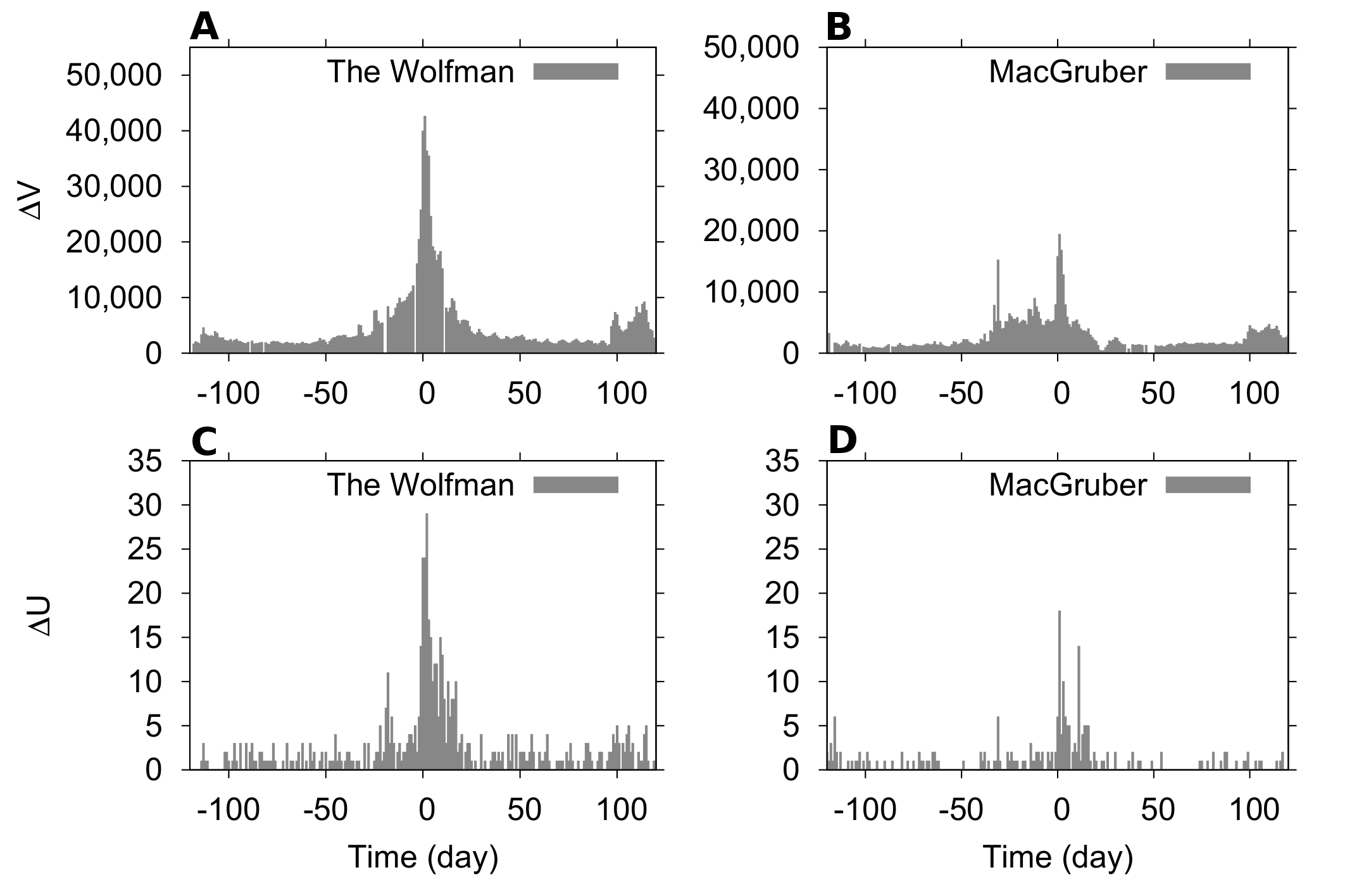

Supplement: Figure S1 — Temporal evolution of Wikipedia-based predictors for two individual movies: The Wolfman (2010) and MacGruber. The daily increments of number of views and number of users are shown for the articles in English Wikipedia that correspond to the two movies. The temporal axis shows movie time, i.e., a time-frame in which corresponds to the release date. The Wolfman earned a box office revenue of $ on the release weekend while MacGruber gained only $. Accordingly, predictor variables take larger values in the case of The Wolfman. (TIFF) [file pone.0071226.s001.tiff]
